# Developing a live cell assay for the centriole-cilium transition in flies

**DOI:** 10.1186/2046-2530-4-S1-P74

**Published:** 2015-07-13

**Authors:** H Roque, J Raff

**Affiliations:** 1 Sir William Dunn School of Pathology, University of Oxford, Oxford, UK

## 

Cilia are essential organelles for organism development and have been linked with several human diseases, so called ciliopathies. A cilium is formed from a mother centriole that extends into a separate membrane compartment at the cell surface. Despite the large number of proteins associated with cilia formation/development the interplay of proteins that allow a centriole to form a cilium are largely unknown. Using the well characterised *Drosophila *sensory organ precursor (SOP) cells as a model, we propose to dissect the molecular pathway of cilia formation with live cell imaging and electron microscopy. SOPs divide in a stereotypical manner to produce four cells, only one of which will form a cilium. We have started by imaging centriole dynamics during the SOP divisions to determine how centrioles behave prior to differentiation and cilium formation. These very early studies reveal that centrioles are highly motile, but are tightly apically constricted in the SOP cells and most of their progeny. Further advances in the methodology will be discussed.

